# HIV-1 Replication in HIV-Infected Individuals Is Significantly Reduced When Peripheral Blood Mononuclear Cells Are Superinfected with HSV-1

**DOI:** 10.1100/2012/102843

**Published:** 2012-09-02

**Authors:** Taneth Yamsuwan, Chintana Chirathaworn, Pokrath Hansasuta, Parvapan Bhattarakosol

**Affiliations:** ^1^Interdisciplinary Program of Medical Microbiology, Graduate School, Chulalongkorn University, Bangkok 10330, Thailand; ^2^Department of Microbiology, Faculty of Medicine, Chulalongkorn University, Bangkok 10330, Thailand

## Abstract

Herpes simplex virus (HSV) can cause generalized infection in human immunodeficiency virus- (HIV-) infected patients leading to death. This study investigated HSV-1 replication in PBMCs from 25 HIV-infected individuals and 15 healthy donors and the effects of HSV-1 superinfection on HIV-1 production. Herpes viral entry mediator (HVEM) receptor on T lymphocytes was also evaluated. Our results confirmed that the number of activated (CD3+ and CD38+) T lymphocytes in HIV-infected individuals (46.51 ± 17.54%) was significantly higher than in healthy donors (27.54 ± 14.12%, *P* value = 0.001) without any significant differences in HVEM expression. Even though the percentages of HSV-1 infected T lymphocytes between HIV-infected individuals (79.25 ± 14.63%) and healthy donors (80.76 ± 7.13%) were not different (*P* value = 0.922), yet HSV-1 production in HIV-infected individuals (47.34 ± 11.14 × 10^3^ PFU/ml) was significantly greater than that of healthy donors (34.17 ± 8.48 × 10^3^ PFU/ml, *P* value = 0.001). Moreover, HSV-1 virions were released extracellularly rather than being associated with the cells, and superinfection of HSV-1 at a multiplicity of infection (MOI) of 5 significantly decreased HIV production (*P* value < 0.001).

## 1. Introduction

Human immunodeficiency virus (HIV) infection is an important public health concern in many countries around the world. The progression of HIV infection depends on the immune status of each individual. When the immune status declines, a number of pathogens can cause opportunistic infections such as *Mycobacterium tuberculosis*, *Mycobacterium avium, Cryptococcus neoformans*, *Pneumocystis carinii*, *Candida albicans*, cytomegalovirus, and herpes simplex virus (HSV) [1].

 HSV is a common human pathogen from the family *Herpesviridae,* subfamily of *Alphaherpesvirinae,* and genus *Simplexvirus* [2]. There are 2 serotypes type 1 (HSV-1) and type 2 (HSV-2). HSV infection causes diseases ranging from asymptomatic to severe symptoms which can result in death. HSV usually causes localized infection but in newborns or immunocompromised patients such as HIV-infected patients, they generally develop severe disseminated disease [3, 4]. A number of studies conducted in HSV-2/HIV-1 coinfected patients have shown that there is a synergistic relationship between the viruses. For example, when HSV-2 is reactivated, this effect also increases HIV-1 viral load in the plasma and genital secretions in both asymptomatic and symptomatic HSV-2 coinfected patients [5, 6], whereas high viral titres of HSV-2 mucosal genital shedding is frequently detected in HIV coinfected patients [7–9]. Therefore, this synergistic relationship between HSV and HIV-1 may enhance HIV-1 disease progression and increase the risk of either HIV-1 or HSV transmission.

HSV generally enters and replicates in lymphocytes and epithelial cells [10, 11]. Growth efficacies of HSV in different cell types have been reported. Epithelial cells, unlike lymphocytic cells, can produce higher amounts of HSV [10]. In order for HSV to enter the cell, many host cell surface receptors are required. For example, herpes viral entry mediator (HVEM), a receptor that restricts expression of immune cells especially of lymphocytes [12, 13], increases when activated with mitogens such as phytohemagglutinin [14]. This activation increases HSV-1 production in the T lymphocytes [14]. Since there are high numbers of activating T lymphocytes in HIV-infected patients [15], hence the hypothesis of this study is that HSV-1 replication in T lymphocytes of HIV-infected patients would produce more HIV than HIV-uninfected patients. Therefore, this study compared the yield of HSV-1 and HIV-1 productions in mock and superinfected peripheral blood mononuclear cells (PBMCs) from healthy blood donors and HIV-infected individuals. The study also evaluated the presence of HVEM on T lymphocytes and the number of activated HSV-1-infected T lymphocytes.

## 2. Materials and Methods

### 2.1. Study Population and Specimen Collection

A total of 40 voluntary subjects, 15 healthy blood donors (control group), and 25 HIV-infected individuals (target group), agreed to join the study. All participants have signed the informed consent which was preapproved by the Ethical Committee for Research, Faculty of Medicine, Chulalongkorn University, Bangkok, Thailand. All HIV-infected individuals with at least one-year history of HIV infection, who attended the Thai Red Cross Anonymous Clinic, were HIV-treatment naïve and had no signs of opportunistic infections. Blood was collected from all participants in 5 mL EDTA and 7 mL heparin vacutainers. Blood was immediately processed after phlebotomy. Plasma was collected from EDTA blood, whereas PBMCs were separated from the heparinized blood.

### 2.2. Preparation of Peripheral Blood Mononuclear Cells (PBMCs)

PBMCs were separated from heparinized blood by density-gradient centrifugation. Briefly, 7 mL of blood was resuspended with equal volume of RPMI 1640 (Gibco, BRL, USA) and overlaid on ficoll-hypaque (density gradient 1.077 g/L), followed by centrifugation at 1,500 rpm for 30 minutes at room temperature. After centrifugation, PBMCs were harvested and washed twice with RPMI 1640 and then resuspended in RPMI 1640 supplemented with 10% fetal bovine serum (FBS; Hyclone, UK) and further processed for analysis.

### 2.3. Cell Culture and Virus

African green monkey kidney (Vero) cells were grown in growth medium (GM; M199 with Earle's salt (Gibco, USA), supplemented with 10% FBS (Hyclone, UK), 10 mM N-2-hydroxyethylpiperazine-N′-2-ethan sulfonic acid (Sigma, USA), 2 mM L-glutamine (Sigma, USA), and antibiotics (100 *μ*g/mL streptomycin, 100 units/mL penicillin G; Dumex, Thailand)). Vero cells were cultured at 37°C. Standard HSV-1 strain KOS was propagated in Vero cells using maintenance medium (MM: same as GM except with 2% FBS). The amount of viruses was determined by plaque titration assay [16].

### 2.4. Detection of HVEM on Activated (CD3+ and CD38+) T Lymphocytes by Flow Cytometry

PBMCs (5 × 10^5^ cells) from either healthy blood donors or HIV-infected patients were first stained with anti-HVEM (Abcam, UK) for 30 minutes at 4°C. These cells were next washed twice with 3 mL of cold washing buffer (Phosphate Buffered Saline (PBS) with 0.5% bovine serum albumin, 0.1% NaN_3_). The cells were then stained with goat anti-mouse-PE (Dako, Denmark) for another 30 minutes at 4°C and washed twice. After HVEM staining process, the cells were further stained with a mixture of anti-CD3-PerCP and anti-CD38-APC (Becton Dickinson; BD, USA) for 30 minutes at 4°C and washed twice. Finally, the cells were resuspended in 500 *μ*L of 1% paraformaldehyde in PBS and analyzed by flow cytometry. The number of cells in each sample was analyzed by FACScan (BD, USA) and calculated by using the Cell-Quest software. The frequency (percentage) was determined by two-color dot plot analysis and density (mean fluorescent intensity; MFI) was determined by utilizing histogram plot. Unstained PBMCs were used as negative control. Cells for negative control were done in parallel with the stained cells.

### 2.5. Analyses of HSV-1 Infected Subpopulation of T Lymphocytes by Flow Cytometry

One million PBMCs from each participant were infected with HSV-1 at multiplicity of infection (MOI) of 5. After 24 hours, 500 *μ*L of 0.02% EDTA were added to the infected cells and incubated at 37°C for another 10 minutes. The cells were washed with 3 mL of cold washing buffer and 500 *μ*L of 4% w/v paraformaldehyde was added and incubated for another 10 minutes. The cells were washed again and 500 *μ*L of FACS permeabilizing solution (BD, USA) was added. The cells were incubated at room temperature in the dark for 30 minutes and washed once. The infected cells were then incubated with rabbit-anti-HSV-1 antibody (Dako, Denmark) at 4°C for 30 minutes, washed once, and incubated with FITC-conjugated swine anti-rabbit antibodies (Dako, Denmark) for 30 minutes. After the cells were indirectly stained for HSV-1 antigens, the cells were further stained with anti-CD3-PerCP, anti-CD4-APC, and anti-CD8-PE for another 30 minutes and washed once. The cells were resuspended in 500 *μ*L of 1% paraformaldehyde and stored overnight at 4°C in the dark for analysis. All monoclonal antibodies were purchased from BD, USA. The number of cells in each sample was analyzed by FACScan (BD, USA).

### 2.6. Inoculation of PBMCs with HSV-1

One million PBMCs from either healthy donors or HIV-infected individuals were infected with HSV-1 at MOI of 5 for 24 hours. The infected cells and supernatant from culture were collected. After centrifugation, the supernatant, containing free virions, was immediately combined and collected into one tube and labeled as cell culture supernatant. The remaining cell pellet was resuspended with 1 mL of MM. This MM has been frozen and thawed at least three times. The cell lysate, containing virions associated cells, was collected by centrifugation at 2,000 rpm for 20 minutes at 4°C. The cell lysate and cell culture supernatant were kept at −70°C. The amount of HSV-1 production was performed by plaque titration assay on both cell lysate and cell culture supernatant while quantification of HIV RNA was done only on cell culture supernatant PBMCs of HIV-infected individuals.

### 2.7. Quantification of HIV

The amount of HIV RNA was quantified by using the ultrasensitive nucleic acid amplification test, AMPLICOR HIV-1 MONITOR Test, v1.5 (Roche Diagnostic, USA), and the standard COBAS AMPLICOR Analyzer (Roche Diagnostic, USA). The procedures for both ultrasensitive and standard methods were followed according to the manufacturers' instructions. The range of both assays was combined together which ranged from <50 to 750,000 copies/mL. HIV was quantified in the plasma of the HIV-infected individuals and cell culture supernatant that was inoculated with HSV-1.

### 2.8. Data Analysis

Data are presented as mean ± SD. The Mann-Whitney *U* test and paired *t-*test were used to determine the statistical significance of the differences observed between both groups. *P* value ≤ 0.05 was considered statistically significant.

## 3. Results

### 3.1. Study Population

There were a total of 25 HIV-infected, antiretroviral naive individuals; 16 males (64%) and 9 females (36%) with the mean age ± SD of 33.92 ± 8.98 (22–62) years were recruited. The number of CD4+ cell count varied between 59–878 cells/mm^3^. Only 3 of them had CD4+ cell count of less than 200 cells/mm^3^. Their HIV-RNA loads on the day of collection ranged from <50 to 194,618 copies/mL (mean ± SD = 38,969.96 ± 54,103.52). In the control group, there were 15 healthy donors; 7 males (46.67%) and 8 females (53.33%) with the age between 21 and 32 years old (mean ± SD = 24.40 ± 2.82). Unfortunately, CD4+ cell count was not determined in the control group.

### 3.2. Determination of HVEM+, CD3+, and CD38+ T Cells

The number of activated T lymphocytes (CD3+ and CD38+) from HIV-infected individuals (mean ± SD = 46.51 ± 17.54%) was significantly higher than those of the healthy donors (mean ± SD = 27.54 ± 14.12%) (*P* value = 0.001). However, the percentages of HVEM+ T cells (mean ± SD = 0.50 ± 0.53%) and HVEM/CD38 coexpressing T lymphocytes (mean ± SD = 0.79 ± 0.92%) from HIV-infected individuals were slightly lower than those of the controls (mean ± SD = 0.57 ± 0.37% and 0.80 ± 0.99%, resp.), but these differences were not statistically significant (Table 1). We then investigated whether the intensity of HVEM expression on the cell surface was different or not between both groups. MFI of HVEM was analyzed, and the results showed that there were no significant differences between the two groups (*P* value = 0.142). It is possible that the status of the HIV-infected individuals may have influenced the number of activated T lymphocytes. However, 3 HIV-infected individuals who had CD4+ cell count of less than 200 cells/mm^3^ also showed the same pattern as those who had CD4+ cell count of more than 200 cells/mm^3^ (data not shown).

### 3.3. HSV-1 Infection in T Cells

When PBMCs of both the control and target groups were infected with HSV-1 at MOI of 5 for 24 hours, the number of HSV-1 infected T lymphocytes (HSV-1+, CD3+) from healthy donors (mean ± SD = 80.76 ± 7.13%) was comparable to HIV-infected individuals (mean ± SD = 79.25 ± 14.63%) as shown in Table 1. Moreover, CD4+ T lymphocytes of HIV-infected individuals (mean ± SD = 97.60 ± 2.20%) seemed to be more susceptible to HSV-1 infection than those of the healthy donors (mean ± SD = 92.93 ± 3.90%, *P* value < 0.001). This susceptibility was also seen in CD8+ T lymphocytes from HIV-infected individuals (97.7 ± 3.62%) which was significantly different to the controls (91.89 ± 9.33%; *P* value = 0.001).

**Figure 1:**
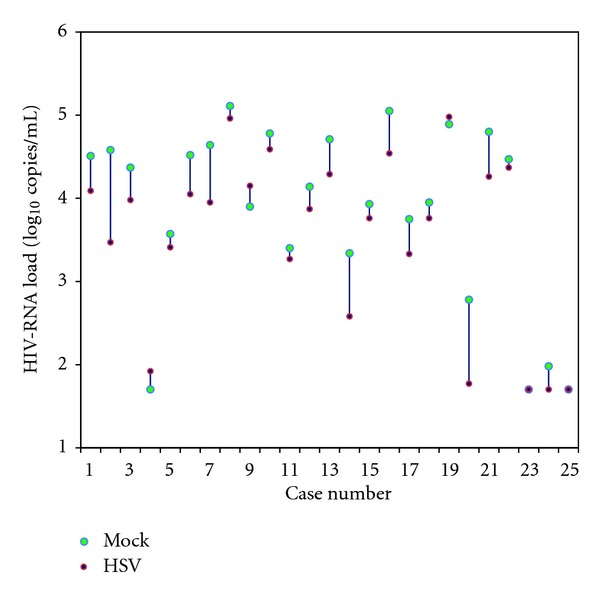
HIV-RNA load obtained from mock and HSV-1 infected PBMCs of 25 HIV-infected individuals.

**Figure 2:**
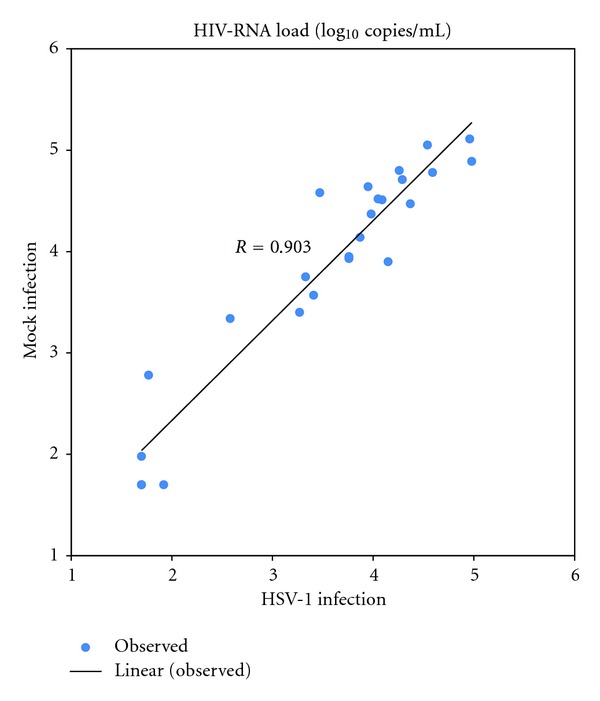
Correlation of HIV-RNA load between mock and HSV-1 infected PBMCs of 25 HIV-infected individuals.

After 24 hours following infection, HSV-1 production in PBMCs from HIV-infected individuals (mean ± SD = 47.34 ±11.14 × 10^3^ PFU/mL) was significantly higher than the healthy donors (mean ± SD = 34.17 ± 8.48 × 10^3^ PFU/mL, *P* value = 0.001). Release of HSV virions extracellularly are characterized in Table 1. From the paired *t*-test, HIV production from mock infection of the cell culture supernatant (3.85 ± 1.09 log_10_ copies/mL) was significantly greater than HSV-1 superinfection (3.54 ± 1.05 log_10_ copies/mL) with a *P* value of < 0.001. According to the Pearson correlation of 0.95 and linear regression of 0.903, in most cases, after HSV-1 infection, HIV-RNA load (20/25, 80%) dramatically declined (Figures 1 and 2). From these 5 cases, only 3 had an increase of HIV-RNA load after HSV-1 infection, whereas in the other two cases, there were no changes in the HIV-RNA load.

## 4. Discussion

In HIV-infected patients, HSV reactivation is frequent and can cause localized and disseminated or systemic infections [3, 4]. The mechanism of disseminated or systemic spread is unclear. According to the results from an *in vitro* study, an increase in the expression of HVEM on activated Jurkat T lymphocytes resulted in higher yield of HSV-1 [14]. This is because HVEM receptor is a member of the tumor necrosis factor superfamily that binds to lymphotoxins, inducible expression, and competes with HSV glycoprotein D for HVEM, a receptor expressed on T lymphocytes (LIGHT). LIGHT is a natural ligand of HVEM and also a tumor necrosis factor superfamily ligand that regulates T-cell immune responses by signaling through the HVEM and the lymphotoxin *β* receptor. As for chronic HSV infection, T lymphocytes of HIV-infected patients are continuously activated [15, 17]. Our results confirm that T cells are constantly activated in HIV-infected patients (Table 1). Therefore, HSV viremia in HIV-infected patients may come from HSV-infected activated lymphocytes.

This suggests that activated T cells of HIV-infected individuals have HVEM receptor. This data also corroborates the findings by Morel et al. [18] that HVEM is constitutively expressed on activated peripheral blood T- and B-lymphocytes which increase the cells' susceptibility to HSV infection. Aside from that, these cells also express LIGHT which has been shown to be associated with activated lymphocytes. However, the results from this *ex vivo* study showed lower HVEM expression on activated T lymphocytes from HIV-infected patients compared to healthy donors but this was not significant (Table 1). One possible explanation for this is that resting T cells have higher levels of HVEM and T cells in HIV-infected patients have lower HVEM expression because they are continuously activated and over time, upon activation, this expression is significantly reduced [18]. Therefore, under continuous stimulation, the number of HVEM molecule may become equivalent to an unstimulated cell because of the formation of the LIGHT-HVEM complex.

Like HVEM expression, there were no differences in the number of HSV-1 infected T-lymphocytes between both groups (Table 1). After evaluating the yield of HSV-1, it was found that HSV-1 production in HIV-infected individuals (47,340 ± 11,140 PFU/mL) was statistically higher than healthy donors (34,170 ± 8,480 PFU/mL, *P* value = 0.001) even though the titer difference was only 0.14 log_10_. This indicated that CD4+ and CD8+ T cells of HIV-infected individuals were more susceptible to HSV infection than the healthy donors. A possible explanation for this is that the HVEM receptor and the number of HSV-1 infected cells could have contributed to this upsurge of HSV-1 production, but this is highly unlikely because there were no elevations of HVEM expression and number of HSV-1 infected cells between both groups. Another possible explanation may lie in the different stages of the cells. Activated cells have more transcription ability [19] than unactivated cells and hence, under such condition, HSV transcription in these activated cells may be more efficient than in unactivated cells. It is also possible that the composition of the cell components in the PBMCs such as B lymphocytes, monocytes, and NK cells may play some role in supporting the growth of HSV-1 [12, 13].

 An alternative explanation for higher levels of HSV in HIV-infected patients is based on HSV's mechanism in releasing its virions. HSV is commonly released from the epithelial cells by cell-to-cell contact resulting in virus associated cells [20]. However, in this study, more than 90% of the HSV-1 virions were released from the PBMCs. This indicated that the circulating white blood cells can directly release HSV-1 virions into the blood stream, hence enhancing the disseminated infection in HIV-infected individuals.

 Other possible explanation may be due to the synergistic relationship between HIV-1 and HSV-2. Nagot et al. [21] showed that HSV-2 coinfected women had significantly higher amounts of HIV-1 in their plasma and genital secretions. Multiple studies have shown that a persistent infection of HSV-2 will increase HIV viral load [22, 23], whereas treatment for HSV-2 reduced HIV-1 by 0.5 log_10_ copies/mL in the plasma [24]. Chapman et al. [25] confirmed the synergistration between HSV immediate early 1 and HIV tat proteins was capable of activating HIV-1 proviral DNA.

Moreover, during HSV-1 and HIV-1 co-infection, pseudovirions can occur [26]. It has been shown that there is an increase in HIV virion production in PBMCs from HIV-infected patients with HSV-1 infection. In contrast, our results showed that HIV-1 production from PBMCs superinfected with HSV-1 was lower than the mock infection by approximately 0.31 log_10_ (Figure 1 and Table 1). According to the Pearson correlation of 0.95 and linear regression of 0.903, HIV-RNA load in 80% (20/25) of the HIV-infected individuals was lower after HSV-1 infection (Figures 1 and 2). This reduction can be explained by the different propagation environments of the cells. In an *ex vivo* environment, cytokines and chemokines that are normally produced by the cells *in vivo* are not present in culture. These naturally occurring cytokines and chemokines have been shown to affect viral production for both HSV and HIV virions [17]. Similar phenomenon was previously observed in HHV-6, a member of *Herpesviridae* which frequently reactivates in HIV-infected patients. Csoma et al. [27] demonstrated that HHV-6 significantly suppressed HIV replication in HHV-6 and HIV coinfected macrophage culture due to increase secretion of RANTES and IL-8.

Another explanation for this is based on the different MOIs used. Calistri et al. [6] previously reported that HSV infection with low MOI enhanced HIV production, whereas a higher MOI suppressed viral production. HSV at MOI of 10 suppressed HIV-1 production, whereas MOI of 1 enhanced HIV replication. Our results are consistent with reports for higher MOIs (data not shown). Unfortunately, this study did not infect the cells using lower MOIs because this study continued to explore the effects of MOI of 5.

Beside this limitation, CD4 count was not obtained in the control group. However it should be noted that the lack of CD4 data in the control group has not affected the results of the study. Another limitation of the study is the use of PBMCs instead of purified T lymphocytes. The synergistic effect between HIV and HSV would be more pronounced if purified T cells were used. It is unclear if the other cells such as T, B, NK, and monocytes have affected the results obtained from this study. Therefore, it is highly recommended that future studies should use purified T cells instead of PBMCs in order to detect the real effect of HSV on HIV. For this study, PBMCs were used to simulate the immune system in real-life situation. Regardless of this, the data from this study is consistent with higher MOIs and therefore has a significant implication for the field of HIV.

 In conclusion, HSV-1 can infect both CD4+ and CD8+ T lymphocytes. HSV-1 replication in these T-lymphocytes is not dependent only on the presence of HVEM receptor. Even though the growth of HSV-1 is higher in PBMCs from HIV-infected individuals, however, its growth decreases the production of HIV virion. HSV release from infected PBMCs may play an important role in disseminated HSV infection in HIV patients.

## Figures and Tables

**Table 1:** Characteristics of PBMCs from HIV-infected individuals and healthy donors.

Experiment	Mean ± SD		
	HIV-infected individuals	Healthy donors	*P* value
	*N* = 25	*N* = 15	
Activated T lymphocytes			
percentage of CD3+ and CD38+	46.51 ± 17.54	27.54 ± 14.12	0.001

HVEM expressing T lymphocytes			
percentage of CD3+HVEM+	0.50 ± 0.53	0.57 ± 0.34	0.282
percentage of CD38+HVEM+	0.79 ± 0.92	0.80 ± 0.99	0.890
MFI of HVEM	3.75 ± 1.57	4.92 ± 2.11	0.142

HSV-1 infected T cells			
percentage of CD3 + HSV-1+	79.25 ± 14.63	80.76 ± 7.13	0.922
percentage of CD4+HSV-1+/Total CD4+	97.60 ± 2.20	92.93 ± 3.90	<0.001
percentage of CD8+HSV-1+/Total CD8+	97.79 ± 3.62	91.89 ± 9.33	0.001

HSV-1 titers (×10^3^ PFU/mL)			
Lysate supernatant (intracellular)	3.54 ± 1.21	2.17 ± 0.90	0.001
Culture supernatant (extracellular)	43.80 ± 11.30	32.00 ± 8.19	0.002
Total amount	47.34 ± 11.14	34.17 ± 8.48	0.001

HIV titers from HIV-infected individuals (log_10_ copies/mL)			
With HSV-1 infection	3.54 ± 1.05		<0.001
Mock infection	3.85 ± 1.09		
